# Correction to “Physicians' Perceptions of Prescribing in Pregnancy and Package Inserts: A Survey of General and Hub Hospitals in Japan”

**DOI:** 10.1111/cts.70662

**Published:** 2026-07-06

M. Goto, N. Yakuwa, and A. Murashima, “Physicians' Perceptions of Prescribing in Pregnancy and Package Inserts: A Survey of General and Hub Hospitals in Japan,” Clinical and Translational Science 19, no. 6 (2026): e70602, https://doi.org/10.1111/cts.70602.

In the article cited above, the institutional affiliation for Atsuko Murashima was incorrect.

The correct affiliation is “Japan Drug Information Institute in Pregnancy, National Center for Child Health and Development.”

We apologize for this error.

